# A Bioinformatics Approach to Prioritize Single Nucleotide Polymorphisms in TLRs Signaling Pathway Genes

**Published:** 2016-05-01

**Authors:** Behnam Alipoor, Hamid Ghaedi, Mir Davood Omrani, Milad Bastami, Reza Meshkani, Taghi Golmohammadi

**Affiliations:** 1 *Department of Biochemistry, Faculty of Medicine, Tehran University of Medical Sciences, Tehran, Iran.*; 2 *Department of Medical Genetics, Faculty of Medicine, Shahid Beheshti University of Medical Sciences, Tehran, Iran.*; 3 *Department of Medical Genetics, Faculty of Medicine, Tabriz University of Medical Sciences, Tabriz, Iran.*

**Keywords:** Bioinformatics; *in-silico* analysis; single nucleotide polymorphisms; toll- like receptors

## Abstract

It has been suggested that single nucleotide polymorphisms (SNPs) in genes involved in Toll-like receptors (TLRs) pathway may exhibit broad effects on function of this network and might contribute to a range of human diseases. However, the extent to which these variations affect TLR signaling is not well understood. In this study, we adopted a bioinformatics approach to predict the consequences of SNPs in TLRs network. The consequences of non-synonymous coding SNPs (nsSNPs) were predicted by SIFT, PolyPhen, PANTHER, SNPs&GO, I-Mutant, ConSurf and NetSurf tools. Structural visualization of wild type and mutant protein was performed using the project HOPE and Swiss PDB viewer. The influence of 5′-UTR and 3′- UTR SNPs were analyzed by appropriate computational approaches. Nineteen nsSNPs in TLRs pathway genes were found to have deleterious consequences as predicted by the combination of different algorithms. Moreover, our results suggested that SNPs located at UTRs of TLRs pathway genes may potentially influence binding of transcription factors or microRNAs. By applying a pathway-based bioinformatics analysis of genetic variations, we provided a prioritized list of potentially deleterious variants. These findings may facilitate the selection of proper variants for future functional and/or association studies.

Toll-like receptors (TLRs) are a major class of the pattern- recognition receptors of the innate immune system involved in the identification of pathogen-associated molecular patterns (PAMPs) from infectious pathogens (1-2). These trans-membrane proteins engage with PAMPs and trigger activation of intracellular signaling cascades, leading to the induction of genes that regulate the expression of pro- inflammatory cytokines and chemokines (3-4). Due to the critical roles of TLRs signaling network in the initiation of innate immune responses, malfunction of genes involved in this pathway may predispose individuals to numerous human diseases ranging from infectious and chronic inflammatory to cancers and autoimmune diseases (5-6).

Accumulating evidence now suggests that genetic variations in TLRs pathway genes may exhibit deleterious effects on gene function, leading to the dysregulation of this signaling pathways (7-8). Single nucleotide polymorphisms (SNPs) are the shortest and the most frequent variations in the human genome. Among these, the functional consequences of untranslated regions (UTRs) and non-synonymous (nsSNPs) SNPs are of special interest, as they can either modulate gene expression or influence protein structure and function (9-10). Although the contribution of SNPs in TLR signaling to human pathological states was addressed by several studies, a comprehensive and prioritized list of SNPs potentially affecting the function and regulation of this pathway is still lacking. Therefore, this study aimed to systematically identify the UTR-SNPs and nsSNPs in genes involved in TLRs signaling network by employing a bioinformatics approach and predicting their deleterious functional and structural consequences.

## Materials and methods

**Retrieving SNPs in TLRs pathway genes**

**Fig. 1:**
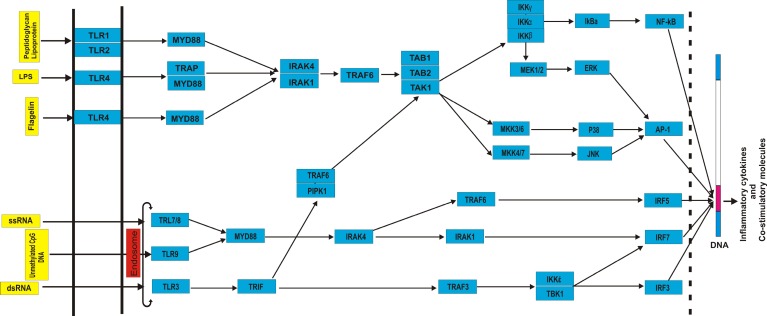
Schematic presentation of gene network implicated in TLR signaling pathway. Direction of signal transduction is exhibited by arrows.

Data on the human TLRs pathway genes were collected from national center for biological information (http://www.ncbi.nlm.nih.gov/) (acce-ssed May 2015) (Table 1). Genes implicated in TLRs pathway and their functional connections were retrieved by querying Kyoto encyclopedia of genes and genomes (KEGG) (http:// www. genome.jp/ kegg/) (accessed May 2015) (Figure 1). SNPs located in TLRs network genes were retrieved from dbSNP (http:// www. ncbi. nlm. nih. gov/SNP/) (accessed June 2015). For each SNP, the following information was recorded: SNP ID, genomic coordinate, and variation type. Protein information of TLR network genes was retrieved from UniProt (http: // www. uniprot.org/) (accessed

June 2015).

**Predicting UTR-SNPs consequences**

To evaluate the conservation score, we used genomic evolutionary rate profiling (GERP) track implemented in UCSC (https://genome.ucsc.edu/) to calculate the GERP++conservation score for each SNPs. Genomic Evolutionary Rate Profiling (GERP) is a method for producing position-specific estimates of evolutionary constraint using maximum likelihood evolutionary rate estimation. Constraint intensity at each individual alignment position is quantified in terms of a "rejected substitutions" (RS) score, defined as the number of substitutions expected under neutrality minus the number of substitutions "observed" at the position. Positive scores represent a substitution deficit (i.e., fewer substitutions than the average neutral site) and thus indicate that a site may be under the evolutionary constraint. Negative scores indicate that a site is probably evolving neutrally; negative scores should not be interpreted as evidence of accelerated rates of evolution because of too many strong confounders, such as alignment uncertainty or rate variance.

The effects of UTR-SNPs on local RNA secondary structure were predicted using mode 1 of RNAsnp program (v 1.1). The software requires RNA sequence and SNP as inputs and uses a window of 400 nucleotides, ±200 nucleotide on either side of the SNP position to obtain subsequences and generate the base-pairing probability matrix for the corresponding wild type and mutant alleles. Then, RNAsnp computes the Euclidian distance (d) and Pearson correlation coefficient (r) for all sequence intervals with a minimum length of 50 that have self-contained base pairs to assess structural difference between the wild type and mutant alleles and reports the interval with the maximum base pairing distance (dmax) or minimum correlation coefficient (rmin) along with the corresponding empirical p-value (11). Here, we used both measures independently and defined structure disruptive UTR-SNPs as those with significant dmax or rmin (significance threshold is p< 0.2 as defined by RNAsnp).

RegulomeDB Version 1.1 (12) was used to annotate UTR-SNPs with known and predicted regulatory elements of the genome including the regions of DNase hypersensitivity, binding sites and motifs of transcription factors, chromatin state and the expression of quantitative trait loci.

To have further annotations, we identified 3'-UTR SNPs residing in microRNAs target sites. A comprehensive dataset of experimentally supported miRNAs target sites, including CLIP-Seq supported interactions from starBase version 2 (http:// starbase.sysu.edu.cn/) (13) and CLASH verified interactions extracted from PolymiRTS database, were compiled (http://compbio.uthsc.edu/miRSNP/) (14).

**Table 1:** TLR signaling pathway genes list.

	**Name**	**Gene ID**	**Location**	**MIM**	**Number of SNPs**
1	TLR1	7096	Chr 4	601194	321
2	TLR2	7097	Chr 4	603028	537
3	TLR3	7098	Chr 4	603029	400
4	TLR4	21898	Chr 9	603030	606
5	TLR5	7100	Chr 1	603031	790
6	TLR6	10333	Chr 4	605403	854
7	TLR7	51284	Chr X	300365	544
8	TLR8	51311	Chr X	300366	270
9	TLR9	54106	Chr 3	605474	509
10	MYD88	4615	Chr 3	602170	123
11	TIRAP	114609	Chr 11	606252	267
12	IRAK1	3654	Chr X	300283	235
13	IRAK4	51135	Chr 12	606883	601
14	TRAF6	7189	Chr 11	602355	579
15	TRAF3	7187	Chr 14	601896	2570
16	TAB1	10454	Chr 22	602615	1989
17	TAB2	23118	Chr 6	605101	3967
18	MAP3K7	6885	Chr 6	602614	1267
19	IKBKG	8517	Chr X	300248	222
20	IKBKB	3551	Chr 8	603258	1376
21	CHUK	1147	Chr 10	600664	750
22	NFKBIA	4792	Chr 14	164008	143
23	NFKB1	4790	Chr 4	164011	2060
24	MAP2K1	5604	Chr 15	176872	2124
25	MAPK1	5594	Chr 22	176948	2335
26	MAP2K3	5606	Chr 17	602315	1329
27	MAP2K7	5609	Chr 19	603014	317
28	MAPK14	1432	Chr 6	600289	1778
29	MAPK8	5599	Chr 10	601158	2450
30	FOS	2353	Chr 14	164810	101
31	TICAM1	148022	Chr 19	607601	438
32	RIPK1	8737	Chr 6	603453	1322
33	IKBKE	9641	Chr 1	605048	696
34	TBK1	29110	Chr 12	604834	895
35	IRF3	3661	Chr 19	603734	199
36	IRF5	3663	Chr 7	607218	284
37	IRF7	3665	Chr 11	605047	173

**Analyzing the functional and structural conse-quences of non- synonymous SNPs**

Phenotypic effects of amino acid substitution on protein function were predicted by Sorting intolerant from tolerant (SIFT) (http://sift.jcvi.org/). In this study, a list of nsSNPs (rsIDs) from NCBI's dbSNP database was submitted as a query sequence to SIFT to predict tolerated and deleterious substitutions for every position of sequence. nsSNPs with SIFT score≤0.05 were classified as deleterious and those>0.05 were classified as tolerated (15).

Polymorphism Phenotyping-2 (PolyPhen-2) (http://genetics.bwh.harvard.edu/ pph2/) predicts possible impact of an amino acid substitution on the structure and function of a human protein using straightforward physical and comparative conside-rations. Input options for this tool are comprised of protein sequence, database ID/ accession number and details of amino acids substitution. For a given substitution, prediction outcome can be one of possibly damaging, probably damaging, and benign (16).

Protein analysis through evolutionary relati-onships (PANTHER) (http:// www.pantherdb. org/) estimates the likelihood of a particular nsSNPs to cause a functional impact on the protein. This tool calculates the substitution position-specific evolutionary conservation (subPSEC) score based on an alignment of evolutionarily related proteins. The subPSEC scores are continuous values from 0 (neutral) to about -10 (most likely to be deleterious). A cutoff of -3 corresponds to a 50% probability that a score is deleterious. From this, the probability that a given variant will cause a deleterious effect on protein function is estimated by Pdeleterious, such that a subPSEC score of -3 corresponds to a Pdeleterious of 0.5 (17).

SNPs database and gene ontology (GO) (http://snps.biofold.org/snps-and-go/snps-and-go.html) have been optimized to predict if a given single point protein variation can be classified as disease associated or neutral. A probability > 0.5 indicates that the mutation at the protein is disease-related (18).

ConSurf web-server (http://consurf.tau.ac.il/) is a bioinformatics tool for estimating the evolutionary conservation of amino acid positions in a protein molecule based on the phylogenetic relations between homologous sequences. The continuous conservation scores are divided into a discrete scale of nine grades for visualization, from the most variable positions (grade 1) colored turqu-oise, through intermediately conserved positions (grade 5) colored white, to the most conserved positions (grade 9) colored maroon.

I-Mutant (http:// folding. uib.es/ i-mutant/ i-mutant 2.0.html) is a neural network based web server for the automatic prediction of protein stability changes upon amino acid substitution. This tool provides the scores for free energy alterations, DDG<0 and DDG> 0 indicate reduction and elevation of the stability, respectively (19).

NetSurfp (http: //www. cbs.dtu. dk/services /NetSurfP/) predicts the relative and absolute surface accessibility and secondary structure of residues in amino acid sequences. The reliability of the surface accessibility prediction is stated in the form of a Z-score, which cannot predict secondary structures of proteins (20).

Project Have your Protein Explained (ProjectHOPE) (http://www.cmbi.ru.nl/hope/home) has been used to study the insight structural features of native protein and the variant models (21). This web server provides three dimensional structural visualization of mutated proteins, and gives the results by using UniProt and DAS prediction servers.

## Results

**SNP** **analysis**

Mining the dbSNP-NCBI and UniProt databases revealed a total of 35802 SNPs in thirty-seven candidate genes in TLRs pathway (Table 2). Among these, 819 and 2502 were located in 5′-UTR and 3′-UTR respectively, and 2172 were identified as nsSNPs.

**Table 2:** Summary results of SNPs mining of candidate genes in TLRs signaling pathway

**Categories**			**Number of SNPs**
Intragenic	exon	Synonymous	1382
		Non-synonymous	2172
	Intron		28654
	Unknown		273
Intergenic	3′-UTR		2502
	5′-UTR		819
Total			35802

**Fig. 2:**
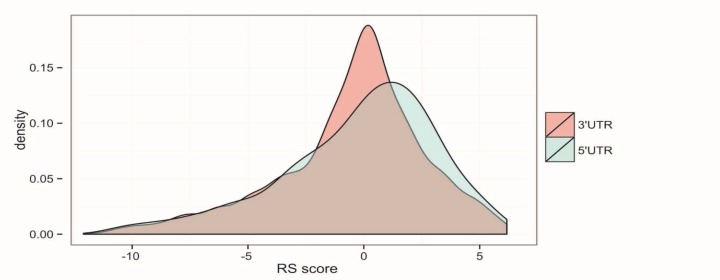
Density plot of GERP++ conservation score (RS score). The figure shows that 5'UTR SNPs have higher (more positive) score than 3'UTR SNPs.

**Fig. 3:**
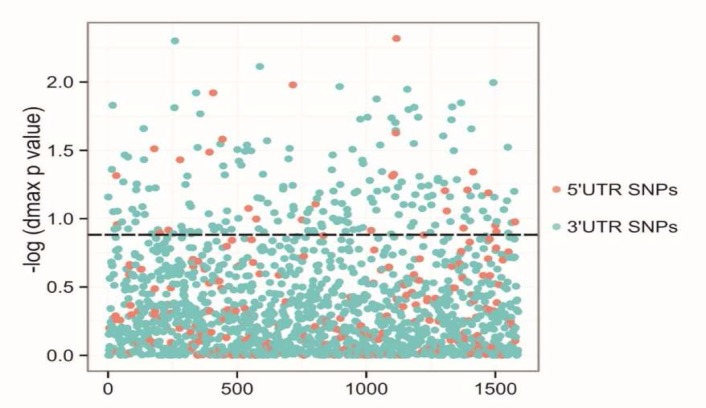
Structure disruptive UTR SNPs in TLR genes. SNPs positioned above dashed line are those with dmax p-value< 0.2, and hence, designated to be structure disruptive.

**Table 3:** Common 3’UTR SNPs resided in miRNA target sites

NFKBIA	hsa-miR-208a-3p	rs696	0.46	**0.07**
MYD88	hsa-miR-520f-3p	rs7744	0.14	0.86
TAB2	hsa-miR-4500	rs7896	0.20	0.27
MAPK14	hsa-miR-4306	rs8510	0.18	0.45
MAPK1	hsa-miR-210-3p	rs9340	0.33	0.21
MAPK1	hsa-miR-186-5p	rs13058	0.04	**0.01**
MAP3K7	hsa-miR-212-3p	rs2131906	0.04	0.38
MAPK14	hsa-miR-381-3p	rs3804451	0.13	0.35
IRAK4	hsa-miR-340-5p	rs4251562	0.04	0.90
MAP3K7	hsa-miR-212-3p	rs9451441	0.01	0.43
TAB2	hsa-miR-33a-5p	rs35859918	0.01	0.47
MAPK1	hsa-miR-217	rs41282607	0.01	**0.08**
TAB2	hsa-miR-539-5p	rs41288431	0.01	0.82
MAPK1	hsa-miR-488-3p	rs61757976	0.01	0.76
TRAF3	hsa-miR-4500	rs72704737	0.29	**0.12**

Target miRNA SNP MAF d_max_ p-value

**Conservation score of UTR SNPs**

We computed GERP++scores for SNPs in UTRs, which represent an evolutionary conservation extent based on alignment of 35 mammals to hg19. Generally, 5′-UTR SNPs were found to be more conserved than 3′-UTR SNPs (Figure 2). With a cut off RS score of ≥ 2, a total of 480 constrained SNPs (including 85 5′-UTR-SNPs and 395 3′-UTR-SNPs) were identified. Moreover, 1200 SNPs (including 141 5′-UTR-SNPs and 1059 3′-UTR-SNPs) were classified as neutrally evolving, which represents a RS score of ≤0. The most conserved SNPs were found in 3′-UTR of *TAB2 *(rs138687718, RS score= 6.17), *MAPK14 *(rs377447706, RS score= 6.17) and *FOS* (rs45480193, RS score= 6.16).

**Influence of UTR-SNPs on RNA secondary structures**

Our analysis showed that 313 UTR-SNPs were structure disruptive as defined by dmax p- value P<0.2 (Figure 3). Considering both dmax and rmin, there were 232 unique structure disruptive UTR-SNPs. The top five genes enriched for structure disruptive SNPs were *MAPK14 *(n= 23), *TLR7* (n= 12), *TLR4* (n= 10), *MAPK1* (n= 10), and *TRAF3* (n= 8). 

**Annotation of SNPs with regulatory elements**

Disease associated variants are enriched in regulatory elements of the genome. Using RegulomeDB, we annotated UTR-SNPs within regulatory elements. 11 UTR-SNPs were associated with transcription factor binding sites (i.e eQTL). These SNPs were found within 3’UTR of *TAB1 *(rs1010169, rs1010170, rs5757650, rs5750822), *RIPK1 *(rs9503383, rs9405606), *IRF5 *(rs752637, rs3807306), *IRAK4 *(rs4251425) and *TLR9 *(rs187084) genes. 

**Identification of SNPs residing in miRNA target sites**

Intersecting 3′-UTR-SNPs with the experimentally validated miRNAs target site datasets, we found 314 SNPs resided in microRNAs target sites. Since miRNA target sites are under selective pressure, we refined SNPs in miRNA target sites by minor allele frequency (MAF) threshold of 0.01** (**Table 3**).**

**Table 4:** List of nsSNPs that predicted to be deleterious by both PolyPhen-2 and SIFT tools

	**Gene** **Symbol**	**SNP**	**Allele**	**AA** **substitution**	**PolyPhen Score**	**PolyPhenPerediction**	**SIFT Score**	**SIFT prediction**
1	CHUK	rs56948661	G>A	P623L	1	P.D	0.01	Damaging
2	CHUK	rs61732515	C>G	Q277H	0.999	P.D	0.00	Damaging
3	CHUK	rs112432667	T>C	E492G	0.954	P.D	0.00	Damaging
4	FOS	rs74685695	T>G	V77G	0.999	P.D	0.01	Damaging
5	IRF5	rs112815033	T>C	L450P	1	P.D	0.01	Damaging
6	IRAK4	rs55944915	G>A	R391H	0.999	P.D	0.01	Damaging
7	IRAK4	rs114820168	C>T	R391C	1	P.D	0.00	Damaging
8	MAP3K7	rs77759048	A>T	W55R	1	P.D	0.00	Damaging
9	TBK1	rs34774243	A>G	K291E	0.997	P.D	0.00	Damaging
10	TBK1	rs55824172	C>T	S151F	0.997	P.D	0.00	Damaging
11	TIRAP	rs74937157	T>C	C134R	1	P.D	0.00	Damaging
12	TLR1	rs5743621	G>A	P733L	0.995	P.D	0.00	Damaging
13	TLR1	rs41311402	A>G	L697S	1	P.D	0.00	Damaging
14	TLR1	rs56205407	A>G	I679T	0.999	P.D	0.00	Damaging
15	TLR1	rs117033348	A>G	L144P	1	P.D	0.04	Damaging
16	TLR2	rs5743706	T>A	Y715N	1	P.D	0.01	Damaging
17	TLR2	rs56303479	T>C	L81P	1	P.D	0.00	Damaging
18	TLR2	rs121917864	C>T	R677W	1	P.D	0.00	Damaging
19	TLR3	rs5743316	A>T	N284I	1	P.D	0.00	Damaging
20	TLR3	rs112666655	T>C	L545P	1	P.D	0.00	Damaging
21	TLR3	rs111488413	C>A	P880Q	1	P.D	0.00	Damaging
22	TLR4	rs77214890	G>T	D181Y	1	P.D	0.00	Damaging
23	TLR4	rs80197996	G>T	L470F	1	P.D	0.03	Damaging
24	TLR4	rs55905951	C>G	A676G	1	P.D	0.00	Damaging
25	TLR4	rs55786277	C>T	R804W	0.999	P.D	0.01	Damaging
26	TLR5	rs5744176	T>C	D694G	1	P.D	0.01	Damaging
27	TLR5	rs78098893	T>C	R752G	0.997	P.D	0.01	Damaging
28	TLR6	rs13102250	A>C	L105W	1	P.D	0.01	Damaging
29	TLR9	rs55881257	G>A	R962C	1	P.D	0.01	Damaging

Abbreviations: P.D; probablydamaging

**Fig. 4:**
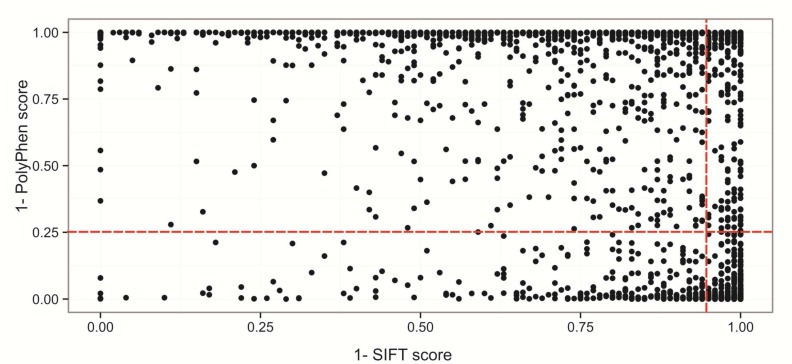
Distribution of SIFT and PolyPhen score of SNPs in coding region. Horizontal and vertical dashed red line correspond to the thresholds for predicting deleterious variants by PolyPhen and SIFT, respectively.

**Prediction of tolerated and deleterious non-synonymous SNPs by SIFT**

SIFT analysis predicted that a total of 785 nsSNPs were damaging (score≤ 0.05) and 1322 nsSNPs had tolerated effects on the candidate genes involved in TLR pathway network (score> 0.05) (Figure 4).

**Prediction of damaging non-synonymous SNPs by PolyPhen-2**

According to our Polyphen-2 results, 610 nsSNPs were predicted “probably damaging”, 353 nsSNPs were predicted “possibly damaging” and 1068 were classified as benign (Figure 4). To increase the accuracy of predictions, results of SIFT and PolyPhen-2 were joined and SNPs with PolyPhen score> 0.95 and SIFT< 0.05 were selected. Accordingly, 29 nsSNPs passed both criteria and were classified as deleterious/damaging (Table 4).

**Prediction of functional impact of non-synonymous SNPs on protein by PANTHER and SNPs & GO.**

According to the PANTHER results, all 29 SNPs possessed the subPSEC score more than −3 and were therefore classified as deleterious (Table 5). As shown in table 5, these SNPs were found to be as disease-associated with the probability >0.5 after analyzing by SNPs & GO.

**Prediction of protein stability analysis by I-Mutant**

According to I- Mutant results, all mutations expect N284I (rs5743316 in *TLR3*), S151F (rs55824172 in *TBK1*) and L105W (rs13102250 in *TLR6*) were predicted to decrease protein stability, with a free energy change value <0.0 (Table 6).

**Prediction of evolutionary conservation of amino acid position by ConSurf**

Our ConSurf analysis revealed that all 29 expected SNPs including the Q277H (*CHUK*), E492G (*CHUK*), L450P (*IRF5*), W55R (*MAP3K7*), K291E (*TBK1*), C134R (*TIRAP*), I679T (*TLR1*), L545P (*TLR3*), R804W (*TLR4*) and R752G (*TLR5*) were located in highly conserved regions and predicted to have functional and structural impacts on TLRs pathway proteins (Table 6).

***In silico*** **solvent accessibility and three-dimensional analyzes of native and mutant protein structures**

By combining the results of SIFT, Poly-phen-2, PANTHER, SNPs & GO, I-Mutant 2.0, and ConSurf servers, 19 mutations were found to be more deleterious in candidate genes. Subsequently, these mutations were analyzed for solvent accessibility and stability, and the results were represented in the following paragraphs (see also Table 7). Visualization of structural features of wild type and mutant protein containing the mentioned deleterious variants was performed using the project HOPE and Swiss PDB viewer.

**Table 5:** PANTHER and SNPs&GO results for prediction of SNPs as disease associated.

			**PANTHER**		**SNPs&GO**		
	**SNPs**	**Substitution**	**subPSEC**	**Pdeleterious**	**Prediction**	**RI**	**Probability**
**1**	rs56948661	P623L	-4.92855	0.87309	Disease	5	0.742
**2**	rs61732515	Q277H	-4.61589	0.83423	Disease	3	0.527
**3**	rs112432667	E492G	-3.99182	0.72945	Disease	4	0.711
**4**	rs74685695	V77G	-4.06862	0.74433	Disease	1	0.545
**5**	rs112815033	L450P	-4.36601	0.79674	Disease	0	0.523
**6**	rs55944915	R391H	-3.64924	0.65684	Disease	0	0.525
**7**	rs114820168	R391C	-4.67097	0.84171	Disease	3	0.643
**8**	rs77759048	W55R	-3.3007	0.57461	Disease	4	0.717
**9**	rs34774243	K291E	-3.56533	0.63768	Disease	5	0.772
**10**	rs55824172	S151F	-4.7119	0.84708	Disease	6	0.804
**11**	rs74937157	C134R	-3.47178	0.6158	Disease	2	0.619
**12**	rs5743621	P733L	-4.51666	0.82005	Disease	2	0.623
**13**	rs41311402	L697S	-4.23845	0.77529	Disease	4	0.712
**14**	rs56205407	I679T	-5.35855	0.91361	Disease	7	0.870
**15**	rs117033348	L144P	-8.17834	0.99439	Disease	5	0.750
**16**	rs5743706	Y715N	-4.34331	0.79303	Disease	4	0.707
**17**	rs56303479	L81P	-6.4936	0.97051	Disease	7	0.855
**18**	rs121917864	R677W	-6.4688	0.96979	Disease	6	0.819
**19**	rs5743316	N284I	-3.91448	0.71392	Disease	5	0.748
**20**	rs112666655	L545P	-4.25641	0.77841	Disease	6	0.823
**21**	rs111488413	P880Q	-8.50881	0.99597	Disease	6	0.811
**22**	rs77214890	D181Y	-4.48068	0.81467	Disease	0	0.511
**23**	rs80197996	L470F	-3.94106	0.71931	Disease	4	0.639
**24**	rs55905951	A676G	-3.16208	0.54043	Disease	0	0.503
**25**	rs55786277	R804W	-5.10263	0.89116	Disease	5	0.748
**26**	rs5744176	D694G	-3.42967	0.6058	Disease	4	0.716
**27**	rs78098893	R752G	-3.16919	0.5422	Disease	2	0.614
**28**	rs13102250	L105W	-5.09383	0.8903	Disease	2	0.583
**29**	rs55881257	R962C	-4.48094	0.81471	Disease	1	0.547

**Table 6:** Summary results of nsSNPs analysis by I-mutant and ConSurf.

				**I-mutant**		**ConSurf**	
	**Gene** **Symbol**	**SNP**	**AA** **substitution**	**DDG (** **Kcal/mol)**	**Stability**	**conservation scale**	**Functional or structural residue**
**1**	CHUK	rs56948661	P623L	-0.97	Decrease	9	F
**2**	CHUK	rs61732515	Q277H	-1.58	Decrease	7	-
**3**	CHUK	rs112432667	E492G	-1.06	Decrease	4	-
**4**	FOS	rs74685695	V77G	-5.25	Decrease	9	S
**5**	IRF5	rs112815033	L450P	-1.74	Decrease	8	-
**6**	IRAK4	rs55944915	R391H	-1.32	Decrease	8	F
**7**	IRAK4	rs114820168	R391C	-0.86	Decrease	8	F
**8**	MAP3K7	rs77759048	W55R	-1.71	Decrease	8	-
**9**	TBK1	rs34774243	K291E	-0.82	Decrease	6	-
**10**	TBK1	rs55824172	S151F	0.01	Increase	9	F
**11**	TIRAP	rs74937157	C134R	-1.55	Decrease	8	-
**12**	TLR1	rs5743621	P733L	-1.33	Decrease	8	F
**13**	TLR1	rs41311402	L697S	-1.51	Decrease	9	S
**14**	TLR1	rs56205407	I679T	-1.91	Decrease	8	-
**15**	TLR1	rs117033348	L144P	-0.79	Decrease	9	S
**16**	TLR2	rs5743706	Y715N	-1.65	Decrease	9	S
**17**	TLR2	rs56303479	L81P	-1.24	Decrease	9	S
**18**	TLR2	rs121917864	R677W	-0.83	Decrease	9	F
**19**	TLR3	rs5743316	N284I	1.23	Increase	9	F
**20**	TLR3	rs112666655	L545P	-1.10	Decrease	7	-
**21**	TLR3	rs111488413	P880Q	-1.26	Decrease	9	F
**22**	TLR4	rs77214890	D181Y	-0.98	Decrease	8	F
**23**	TLR4	rs80197996	L470F	-0.86	Decrease	9	S
**24**	TLR4	rs55905951	A676G	-1.19	Decrease	9	S
**25**	TLR4	rs55786277	R804W	-0.54	Decrease	6	-
**26**	TLR5	rs5744176	D694G	-1.31	Decrease	9	F
**27**	TLR5	rs78098893	R752G	-1.49	Decrease	7	-
**28**	TLR6	rs13102250	L105W	0.91	Increase	9	S
**29**	TLR9	rs55881257	R962C	-2.62	Decrease	8	F

Abbreviations: DDG; free energy change value (DDG<0: Decrease Stability, DDG>0: Increase Stability). The pH and the temperature were set to7 and 25˚C for all submissions, respectively. F: functional residue; S: structural residue.

The rs56948661 in *CHUK *gene leads to P623L. The residue is located on the surface of the protein and mutation of this residue can disturb the interactions with other molecules or other parts of the protein. Moreover, the mutation can disturb the special backbone conformation induced by proline. Conversion of V77G (rs74685695 in *FOS*) causes some structural changes in protein. Glycine residue is smaller than valine and this may lead to loss of the interactions. Furthermore, the mutant residue is more hydrophobic and flexible and can disturb the required rigidity of the protein on this position. For rs114820168 in *IRAK4*, the wild-type (arginine) and mutant (cysteine) amino acids differ in size, hydrophobicity and charge. The difference in charge will disturb the ionic interactions of the wild type residue with D388, E389 and D398. R391H is annotated with rs55944915 in dbSNP database. According to the PISA-database, the mutated residue is involved in a multimer contact. The new residue might be too small to make multimer contacts. In S151F variant, rs55824172 of *TBK1* gene, the mutant residue (phenylalanine) is bigger and more hydrophobic than the wild-type (serine). This conversion will cause the loss of hydrogen bonds in the core of the protein resulting in the disruption of correct folding. 

**Fig 5:**
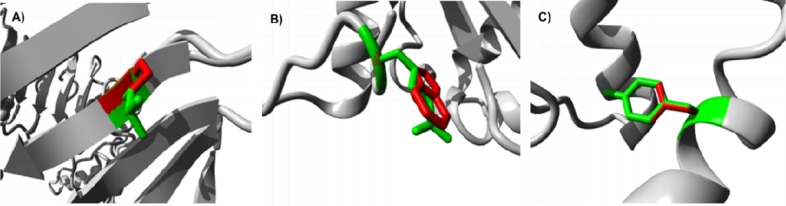
Deep view of superimposed structure of wild and mutant TLR2. A: L81P; B: R677W and C: Y715N. The protein and the side chains of the wild-type and the mutant residue are shown and colored grey, green and red, respectively.

We found that three SNPs in *TLR1*, including P733L (rs5743621), L697S (rs41311402) and L144P (rs117033348), were located in highly conserved regions and predicted to have functional and structural impacts on proteins. For P733L, the mutant residue (leucine) is bigger than the wild-type (proline) and is located on surface of the protein, potentially disturbing its interactions. For L697S and L144P, the mutant residues are smaller than the wild-type residues and will cause an empty space in the core of the protein. In addition, all three mutations are predicted to have functional and structural influences on TLR2 protein (Figure 5).

**Fig 6:**
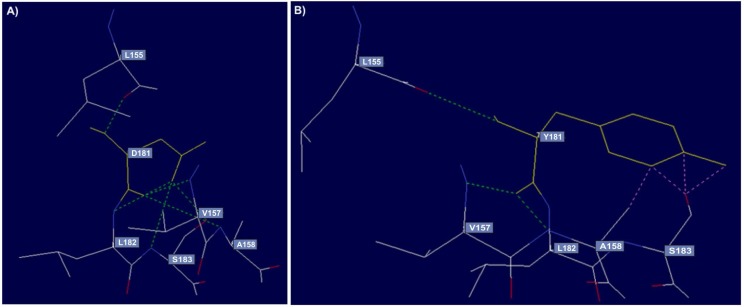
Hydrogen bonding interactions and clashes of wild type and mutant TLR4 at position 181. A: the wild-type residue (D) forms hydrogen bonds (green discontinuous line) with L155, V157, A158, L182 and S183; B: substitution of this amino acid with tyrosine will cause loss of hydrogen bonds with A158, L182 and S183. Moreover, the mutation showed a network of clashes (pink discontinuous line) with A158 and S183 residues.

**Supplementary Table 1:** Surface accessibility of wild-type and mutant variants in TLRs network intermediate molecules.

	**Gene** **Symbol**	**SNP**	**AA** **substitution**	**Class assignment**	**AA**	**AA** **position**	**RSA**	**ASA**	**Z-fit score**
1	CHUK	rs56948661	P623L	Exposed Exposed	P L	623 623	0.544 0.537	77.179 98.306	1.190 1.088
2	FOS	rs74685695	V77G	Buried Buried	V G	77 77	0.082 0.159	12.58 12.55	-0.799 -0.920
3	IRAK4	rs55944915	R391H	Exposed Exposed	R H	391 391	0.500 0.520	114.40 94.58	-0.611 -0.727
4	IRAK4	rs114820168	R391C	Exposed Exposed	R C	391 391	0.500 0.477	114.40 67.04	-0.611 -0.891
5	TBK1	rs55824172	S151F	Buried Buried	S F	151 151	0.132 0.116	15.52 23.30	0.068 -0.048
6	TLR1	rs5743621	P733L	Exposed Exposed	P L	733 733	0.575 0.569	81.57 104.23	0.687 0.717
7	TLR1	rs41311402	L697S	Buried Buried	L S	697 697	0.028 0.030	5.05 3.49	0.951 0.649
8	TLR1	rs117033348	L144P	Buried Buried	L P	144 144	0.038 0.035	6.92 5.023	0.503 0.657
9	TLR2	rs5743706	Y715N	Buried Buried	Y N	715 715	0.152 0.153	32.46 22.39	0.193 0.253
10	TLR2	rs56303479	L81P	Buried Buried	L P	81 81	0.038 0.029	6.93 4.10	0.362 0.758
11	TLR2	rs121917864	R677W	Buried Buried	R W	677 677	0.243 0.255	55.60 61.32	-0.079 -0.088
12	TLR3	rs5743316	N284I	Buried Buried	N I	284 284	0.083 0.088	12.16 16.33	-1.686 -1.081
13	TLR3	rs111488413	P880Q	Exposed Exposed	P Q	880 880	0.401 0.446	56.88 79.65	0.150 0.108
14	TLR4	rs77214890	D181Y	Buried Buried	D Y	181 181	0.240 0.258	34.52 55.24	0.528 0.277
15	TLR4	rs80197996	L470F	Buried Buried	L F	470 470	0.090 0.089	16.40 17.88	0.080 0.247
16	TLR4	rs55905951	A676G	Buried Buried	A G	676 676	0.033 0.034	3.62 2.71	-0.046 -0.158
17	TLR5	rs5744176	D694G	Buried Buried	D G	694 694	0.164 0.173	23.57 13.64	-0.270 -0.384
18	TLR6	rs13102250	L105W	Buried Buried	L W	105 105	0.030 0.031	5.51 7.40	0.843 0.799
19	TLR9	rs55881257	R962C	ExposedExposed	R C	962 962	0.419 0.464	95.95 65.20	0.066 0.045

Abbreviations: RSA: Relative Surface Accessibility; ASA: Absolute Surface Accessibility. Values for wild type and mutant variants are presented by red and green color respectively

For L81P (rs56303479),because this residue is part of some interpro domains like leucine-rich repeat, typical subtype, the interaction between these domains could be disturbed by the mutation. The R677W (rs121917864) mutation leads to substitution of arginine by a bigger and more hydrophobic residue named tryptophan. The difference in charge will disturb the ionic interaction made by the arginine with E649 and 656. The third mutation of *TLR2* occurs at position 715 (rs5743706). The hydrophobicity of the wild-type (tyrosine) and mutant residue (asparagine) differs and the mutation will cause the loss of hydrophobic interactions in the core of the protein. Finally, the size difference between residues makes that the new residue is not in the correct position to make the same hydrogen bond with S646, as the wild-type residue does. For N284I (rs5743316, in *TLR3*), due to the difference in hydrophobicity index of residues, the mutation will cause the loss of hydrogen bonds in the core of the protein and may lead to incorrect folding of protein. The second mutation of *TLR3* (rs111488413) causes P880Q. This mutant residue is bigger than the wild-type residue and can disturb the protein interactions. Additionally, the hydrophobicity of the residue differs; hence, the mutation may cause the loss of hydrophobic interactions.

Concerning D181Y mutation in *TLR4* (rs77214890), the difference in charge will disturb the ionic interaction made by the original residue with R234. Moreover, the hydrophobicity of the native and mutant residue differs. Therefore, this mutation causes the loss of hydrogen bonds in the core of the protein leading to disruption of the correct folding (Figure 6). For rs80197996 (L470F) in *TLR4*, the mutant residue (phenylalanine) is bigger and probably will not fit to bury in the core of the protein. In A676G (rs55905951), the mutant residue is smaller than the wild-type residue. This will cause a possible loss of external interactions. Furthermore, the mutation may cause the loss of hydrophobic interactions with other molecules on the surface of the protein.

Concerning rs5744176 (D694G) of *TLR5*, the wild-type residue forms a salt bridge with K692, R752 and K753. The difference in charge will disturb these ionic interactions. Moreover, the aspartic acid forms a hydrogen bond with N726, but due to difference in hydrophobicity, the mutation causes the loss of hydrogen bond. For the L105W (rs13102250) in *TLR6*, the wild-type (leucine) and mutant (tryptophan) amino acids differ in size. The wild-type residue was buried in the core of the protein, but the mutant residue is bigger and probably will not fit. For rs55881257 (R962C in *TLR9*) the charge of the wild-type residue will be lost; this can cause the loss of interactions with other molecules or residues. Furthermore, this mutation introduces a more hydrophobic residue at this position, probably resulting to loss of hydrogen bonds.

## Discussion

TLRs signaling pathway plays a key role in the host innate immune response. Increasing evidence has suggested that functional SNPs of genes related to TLRs pathway may contribute to diseases ranging from chronic inflammatory to cancers. Since SNPs are the most common genetic variations in human genome, it is expected that genes involved in TLRs pathway contains numerous SNPs. Nevertheless, discriminating deleterious SNPs with potential effects on disease susceptibility from tolerated variants is a major challenge. Therefore, a comprehensive study that systematically analyzes the effects of such SNPs can cost-effectively prioritized SNPs for further analyzes.

*In-silico* analysis of the deleterious effects of SNPs may help to improve our understanding on the biological pathways (22). In this study, we systematically analyzed the SNPs in different parts of genes (5′-UTR, 3′-UTR and coding) in TLRs pathway. A report has suggested that mutation effect prediction algorithms have their own strengths and weaknesses, and therefore, implementing a combination of these tools may help to enhance the accuracy of effect predictions (23). In the present study, we combined the results of the SIFT, PolyPhen, PANTHER, SNPs & GO, I-Mutant and ConSurf algorithms to prioritize the damaging nsSNPs and increase the analysis accuracy. Accordingly, we were able to identify several potentially deleterious nsSNPs in TLRs pathway genes. These SNPs, to the best of our knowledge, have not yet been investigated and therefore may be considered as candidates for association with diseases. These results may pave the ground for future functional and/or association studies and facilitate the process of choosing functional variant for further analyses. 

UTR-SNPs play important roles in gene regulation and accumulating evidence has indicated their contribution to different diseases. Sequence alteration in these regulatory elements has been shown to interfere with transcription factors or microRNA binding, leading to gene dysregulation (24-25). By applying a bioinformatics approach, we evaluated such effects of UTR-SNPs on TLRs pathway genes and identified numerous disease-associated variants that potentially confer the disease risk through affecting transcription factors or miRNAs binding. *TLR9* rs187084, a UTR-SNP which probably interferes with transcription factors binding, has been shown to modify susceptibility to diseases specially renal transplant recipients and cancers (26-27). Several genes of TLRs pathway are regulated post-transcriptionally by miRNAs (28). Our analysis revealed that several SNPs of TLRs network resided in microRNA target sites (Table 3) that may potentially modify miRNA-mediated regulation of these genes. For instance, rs7744 in 3′-UTR of *MYD88* and rs696 in 3′-UTR of *NFKBIA* genes could disrupt the binding of miR-520f-3p and miR-208a-3p, respectively. Matsunaga et al. showed that homozygous minor allele of rs7744 is associated with the severity of ulcerative colitis (29). Moreover, it has been shown that rs696 G>A is associated with the susceptibility to different diseases including coronary artery disease and Behçet's disease (30-31). 

In conclusion, the current study reports the first pathway-based bioinformatics analysis of SNPs in TLRs pathway genes and provides a prioritized list of functional SNPs potentially affecting regulation and function of the pathway. However, we noticed that the complexities of biological pathways merit the need for more experimentation to validate the true effect of these SNPs on TLRs network. Although the functional significance of the candidate SNPs was not experimentally assessed in this study, we believe that our results will help researchers interested in the roles of SNPs in TLRs pathways genes to focus on proper candidate variants. 
